# Enzymatically prepared alginate oligosaccharides improve broiler chicken growth performance by modulating the gut microbiota and growth hormone signals

**DOI:** 10.1186/s40104-023-00887-4

**Published:** 2023-07-03

**Authors:** A La Teng Zhu La, Yuqing Feng, Die Hu, Yimei Feng, Xiaolu Jin, Dan Liu, Yuming Guo, Gong Cheng, Yongfei Hu

**Affiliations:** 1 State Key Laboratory of Animal Nutrition, College of Animal Science and Technology, China Agricultural University, Beijing, 100193 China; 2 State Key Laboratory of Biochemical Engineering, Institute of Process Engineering, Chinese Academy of Sciences, Beijing, 100190 China

**Keywords:** Acetate, Alginate lyases, Alginate oligosaccharides, *Dorea* sp., Gut microbiota

## Abstract

**Background:**

Alginate oligosaccharide (AOS) holds great potential as a novel feed supplement in farm animals. However, the effects of AOS on chicken health and the underlying mechanisms are not fully understood. This study aimed to optimize the enzymatic preparation of AOS by using bacterial alginate lyases expressed in yeast, investigate the effects of the prepared AOS on the growth performance and gut health of broiler chickens, and reveal the underlying mechanisms.

**Results:**

Five alginate lyases from bacteria were cloned into *Pichia pastoris* GS115 and the alginate lyase PDE9 was expressed at relatively high yield, activity and stability in *P. pastoris*. Animal trials were carried out using 320 1-day-old male Arbor Acres broilers (four groups; 8 replicates/group × 10 chicks/replicate) receiving either a basal diet or the same diet supplemented with 100, 200 and 400 mg/kg PDE9-prepared AOS for 42 d. The results showed that dietary supplementation of 200 mg/kg AOS displayed the highest activity in promoting the birds’ ADG and ADFI (*P* < 0.05). AOS ameliorated the intestinal morphology, absorption function and barrier function, as indicated by the enhanced (*P* < 0.05) intestinal villus height, maltase activity, and the expression of *PEPT*, *SGLT1*, *ZNT1*, and occludin. AOS also increased serum insulin-like growth factor-1, ghrelin (*P* < 0.05), and growth hormone (*P* < 0.1). Moreover, the concentrations of acetate, isobutyrate, isovalerate, valerate, and total SCFAs in cecum of birds fed AOS were significantly higher than the control birds (*P* < 0.05). Metagenomic analysis indicated that AOS modulated the chicken gut microbiota structure, function, and microbial interactions and promoted the growth of SCFAs-producing bacteria, for example, *Dorea* sp. 002160985; SCFAs, especially acetate, were found positively correlated with the chicken growth performance and growth-related hormone signals (*P* < 0.05). We further verified that AOS can be utilized by *Dorea* sp. to grow and to produce acetate in vitro.

**Conclusions:**

We demonstrated that the enzymatically produced AOS effectively promoted broiler chicken growth performance by modulating the chicken gut microbiota structure and function. For the first time, we established the connections among AOS, chicken gut microbiota/SCFAs, growth hormone signals and chicken growth performance.

**Graphical Abstract:**

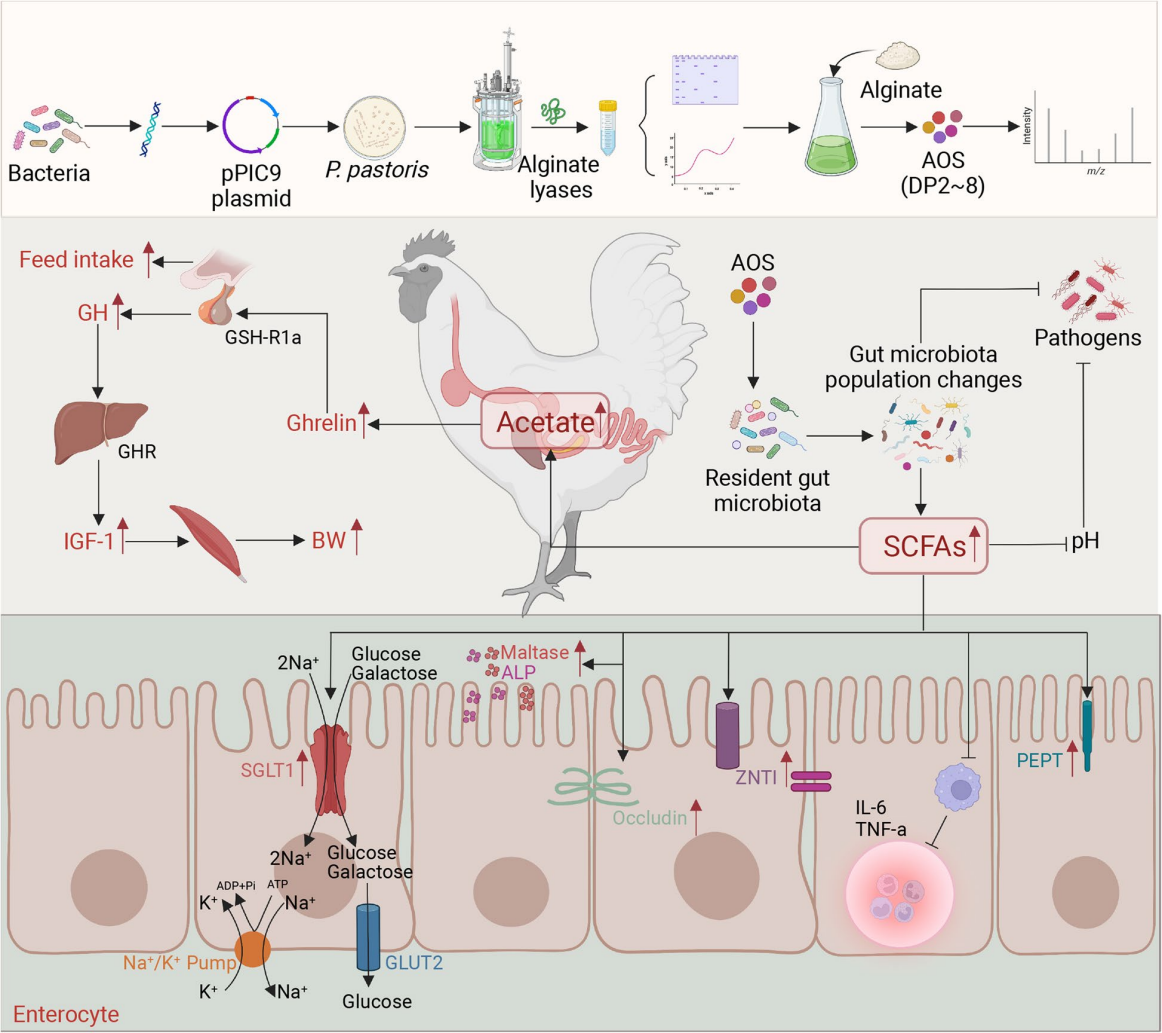

**Supplementary Information:**

The online version contains supplementary material available at 10.1186/s40104-023-00887-4.

## Introduction

Prebiotics are nondigestible carbohydrates that have been proven to not only selectively affect gut bacteria but also regulate host immunity and metabolism in both humans and animals [1]. Different prebiotics, including mannan oligosaccharide (MOS), galacto-oligosaccharides (GOS), fructo-oligosaccharides (FOS), and alginate oligosaccharide (AOS), have attracted more attention for their helpful roles in improving host health in recent decades [2, 3]. Prebiotics are known to influence gut microbial community composition by increasing traditional probiotics especially *Lactobacillus* and *Bifidobacterium* [2]. However, other gut beneficial bacteria, for example, the short-chain fatty acid (SCFA)-producers such as *Alistipes, Faecalibacterium,* and *Dorea* were also found stimulated by the presence of different prebiotics. *Dorea*, members in which are important acetic acid-producers [4], was demonstrated to be promoted by fermentation of the human fecal microbiota on FOS [5], and *Dorea longicatena* was found increased in mice that were transplanted with a human microbiota and fed FOS [6].

Among the well-recognized prebiotics, AOS is produced by alginate, which is a water-soluble, nontoxic compound that possesses various beneficial bioactivities, such as antitumor, immunomodulatory, anti-inflammatory, antioxidant, antimicrobial, prebiotic, and antidiabetes activities [7]. AOS can be prepared from alginate by acid hydrolysis, oxidative degradation, enzymatic degradation and fermentation. Compared with physical and chemical methods, enzymatic methods have advantages in cost, reaction conditions, yield, specificity on substrate and selectivity on products and are regarded as the most promising method for AOS preparation.

The beneficial role of AOS as a feed additive has been demonstrated in different animals. AOS significantly increases the average daily gain (ADG) of weaned pigs, improves their intestinal morphology, digestion-absorption and barrier function, and elevates their antioxidant capacity and serum hormone levels [8, 9]. Besides, AOS modifies the composition of the pig intestinal microbiota and increased the concentrations of SCFAs, which contributes to the intestinal barrier integrity by regulating the NF-κB and AMPK pathways [10]. Additionally, AOS improves the growth performance of juvenile grass carp [11] and modulates the gut microbiota of different aquatic animals [12]. These results suggest that AOS is an effective dietary ingredient to enhance animal growth and health. However, in poultry, although Yan et al. [13] showed that dietary AOS reduced *Salmonella* colonization and promoted the growth of lactic acid bacteria in the broiler chicken cecum, relatively limited studies have been conducted to explore the effects of AOS on bird performance and health.

In this study, we aimed to clone and express alginate lyases from different bacteria in *Pichia pastoris* to optimize the enzymatic preparation of AOS and investigate the role of the enzymatically prepared AOS in improving broiler chicken growth performance and gut health with an emphasis on its effects on gut microbiota structure and function. We also aimed to explore the relationships among AOS, gut microbes, and chicken growth hormone signals to explain the underlying mechanisms.

## Materials and methods

### Cloning and expression of alginate lyases in *P. pastoris* GS115

The coding sequences of five alginate lyases from *Saccharophagus degradans* [14], *Microbulbifer* sp. Q7 [15], *Flammeovirga* sp. NJ-04 [16], *Alteromonas* sp. S89 [17] and *Flammeovirga pacifica* WPAGA1 [18] were commercially synthesized (Beijing Tsingke Biotechnology Co., Ltd., China) and cloned into the pPIC9 plasmid. To facilitate the expression of the recombinant alginate lyase protein in *P. pastoris*, the amino terminal substrate-binding domain of alginate lyases was eliminated, and five protein sequences named PDE9, PDE26, PDE27, PDE28, and PDE29 were generated. The plasmids harboring the cloned genes were transformed into *P. pastoris* GS115 [19]. To induce the expression of the enzyme proteins, the recombinant *P. pastoris* strains were first grown in BMGY (buffered glycerol complex medium), and then transformed into BMMY (buffered methanol-complex medium) and incubated at 30 °C and 250 r/min for 72 h with a final concentration of 1% methanol added every 24 h [20].

### Enzyme assay of alginate lyase

The dinitrosalicylic acid method was used to analyze alginate lyase activity [21]. The optimum pH was determined for each enzyme under standard assay conditions within a pH range of 5–10. The effect of temperature on enzyme activity was investigated with varying temperatures (30–70 °C) at optimal pH. For thermostability analysis, the enzymes were incubated at different temperatures (30–70 °C) for 60 min, and the residual enzyme activity was measured under the optimal pH and temperature.

### Preparation and structural characterization of AOS

Approximately 600 μL of crude alginate lyase was mixed with 100 mL of 10 g sodium alginate (a mixture extracted from *Macrocystis pyrifera* and *Ascophyllum nodosum*, Shandong Jiejing Group Corporation, China) and incubated at 40 °C and 500 r/min for 48 h. After incubation, the mixture was boiled at 60 °C for 60 min and then centrifuged to remove the debris. Samples were obtained from the supernatants by freeze drying and were analyzed by HPLC‒MS.

### In vivo study

#### Ethics statement

All experiments involving animals were conducted according to the ethical policies and procedures approved by the Animal Care and Use Committee of China Agricultural University (Approval no. Aw32902202-1–1).

#### Birds, experimental design and diets

A total of 320 one-day-old male Arbor Acres broilers with an initial average body weight (BW, 43.11 ± 0.26 g) were selected and divided into 4 dietary treatments: (1) basal diet (control, CON); (2) basal diet + 100 mg/kg AOS; (3) basal diet + 200 mg/kg AOS; and (4) basal diet + 400 mg/kg AOS. Each treatment was replicated 8 times, with 10 birds per replicate cage. All birds were provided feed and water ad libitum. All diets were formulated to meet the NRC (1994) [22] and Chinese chicken feeding standards (NY/T-33–2004) [23] (Table 1).

**Table 1:** Basal diet composition (as-fed basis)

Ingredient, %	d 1–21	d 22–42
Corn	56.05	57.35
Soybean meal (CP > 44%)	35.67	32.21
Soy oil	2.60	4.30
Corn gluten meal (CP > 51.3%)	2.00	2.60
Limestone	0.80	0.99
Dicalcium phosphate	1.90	1.44
Salt	0.30	0.30
Methionine (99%, *DL*-form)	0.22	0.12
Choline (50%)	0.20	0.30
Lysine-HCl (78%, *L*-form)	-	0.13
Vitamin premix^a^	0.02	0.03
Mineral premix^b^	0.20	0.20
Ethoxyquin (66%)	0.03	0.03
AOS premix^c^	0.00	0.00
Total	100.00	100.00
Calculated composition^d^, %		
Crude protein	21.54	20.00
Metabolizable energy, kcal/kg	2,970.00	3,070.00
Calcium	1.00	0.90
Available phosphorous	0.51	0.40
Lysine	1.21	1.10

^a^Provided per kg of diet: vitamin premix (1 kg) contained the following: vitamin A, 50 MIU; vitamin D_3_, 12 MIU; vitamin K_3_, 10 g; vitamin B_1_, 10 g; vitamin B_2_, 32 g; vitamin B_12_, 0.1 g; vitamin E, 0.2 MIU; biotin, 0.5 g; folic acid, 5 g; pantothenic acid, 50 g; niacin, 150 g

^b^Provided per kg of diet: copper, 4 g; zinc, 90 g; iron, 38 g; manganese, 46.48 g; selenium, 0.1 g; iodine, 0.16 g; cobalt, 0.25 g

^c^Alginate oligosaccharide premix was added at the expense of corn to supply 0, 100, 200 or 400 mg AOS/kg diet

^d^Calculated value based on the analyzed data for the experimental diets

A double-layer, three-dimensional chicken coop was used, and the birds were reared at stocking density (ranging was 14.3 birds/m^2^). Temperature was gradually decreased by 2 °C per week from 33 °C on d 0 to 21 °C on d 42 and was kept constant thereafter. Birds were immunized as per commercial practice. The indexes of humidity, temperature, light, and hygiene in the chicken house accord with the hygienic requirements of broilers (GB 14925–1994) [24].

#### Growth performance

The individual BW (g) of birds and feed intake (g) by cage were determined at d 21 and 42. Average daily feed intake (ADFI), ADG, and the feed/gain ratio (F/G) were calculated for further analysis. After dosage selection, we focused on 200 mg/kg AOS to reveal its effects on chicken physiology and gut microbiota in the following analyses.

#### Sample collection

Blood samples were collected from the wing (16 per treatment, 2 per replicate close to the average BW of the treatment) at d 41. Serum was obtained with the blood centrifuged at 3,000 × *g* for 10 min at 4 °C and then stored at −20 °C for further analyses. The birds were euthanized by cervical dislocation at d 42. Samples (approximately 2 cm) of the midpoint of the jejunum was fixed in 4% paraformaldehyde and stored at 4 °C for morphological measurements. The jejunal mucosa was scraped with a glass slide, and jejunal tissue was harvested aseptically, and stored at −80 °C for further analyses. Cecal digesta and tonsils were immediately transferred to liquid nitrogen and kept in a −80 °C freezer until microbial population determination and gene expression analysis.

#### Intestinal morphology analysis

The preserved gut segments were dehydrated through a graded ethanol series, embedded in paraffin wax and cut into 5 μm thick cross-sections by using a microtome, and then stained with hematoxylin and eosin (H&E) [25]. In each jejunum sample, all well-oriented villus lengths and their adjacent crypt depths were determined using light microscopy (Leica DM750, Wetzlar, Hessian, Germany) and measured with a Digital Image Processing and Analysis System.

#### Intestinal brush border enzyme activity measurements

Frozen mucosa samples were homogenized with ice-cold physiologic saline at a 1:9 ratio (w/v), and the supernatant was centrifuged at 3,000 × *g*, 4 °C for 15 min [9]. The supernatant protein concentration was assayed using a protein quantification kit (Beyotime Biotechnology, Nantong, Jiangsu, China), and the sucrase, maltase, alkaline phosphatase (ALP), Na^+^/K^+^-ATPase activities in the supernatant were determined using commercial kits (Nanjing Jiancheng Bioengineering Institute, Jiangsu, China) according to the manufacturer's instructions. Briefly, samples, standard, and chromogenic agent were successively added and incubated in the 96 wells microplate, and the optical density values were read on a microplate spectrophotometer (Epoch 2, BioTek Instruments, Inc., Winooski, Vermont, USA) at corresponding wavelength.

#### Total RNA isolation and quantitative real-time PCR

Total RNA extraction, RNA reverse transcription, and real-time fluorescence quantification PCR were carried out with TaKaRa (Takara Biomedical Technology Co., Ltd., Beijing, China) regents according to the manufacturer's instructions. In brief, total RNA was isolated from jejunum and cecal samples using TRIZOL reagent and then treated with DNase I to remove trace DNA. RNA was reverse transcribed to cDNA using the High-Capacity cDNA Reverse Transcription Kit. The real-time quantitative PCR reaction was performed on QuantStudio 7 Flex Real-Time PCR System (Applied Biosystems, Foster City, CA, USA), using TB Green Premix Ex Taq II (Tli RNaseH Plus) with a 10 µL system including 5 µL TB Green Premix, 0.8 µL of primer (forward and reverse primers were premixed), 0.2 µL of ROX Reference Dye II (50 ×), 1 µL of cDNA template, and 3 µL of DNase Free dH_2_O [8, 26]. The primers were commercially synthesized (Invitrogen, Shanghai, China) and are presented in Table S1. Relative gene expression was analyzed by 2^−ΔΔCt^ method with normalization against the *β-actin*.

#### Determination of cecal digesta SCFAs

SCFAs of cecal digesta were quantified using a gas chromatograph (Agilent 5975C GC system, Wilmington, NC, USA) as described by Calik and Ergün [27]. Briefly, the SCFAs of cecal digesta were extracted by double-distilled water and ice-cold 25% (w/v) metaphosphoric acid solution was added into the extracts at a ratio of 1:5 (w/v). Solution placed in ice water for 30 min immediately, homogenized with intermittent vortexing, and then centrifuged for 10 min at 12,000 × *g* at 4 °C. Ultimately, the extracted sample solution was applied to measure the SCFAs content using a gas chromatograph.

#### Serum parameters assay

Following the manufacturer’s instructions, serum ghrelin, insulin-like growth factor-1 (IGF-1), and growth hormone (GH) levels were assayed using commercially available chicken ELISA kits (Angle Gene Biotechnology Co., Ltd., Nanjing, Jiangsu, China). In principle, the wells of the microtiter plates were pre-coated with the antibody (anti-ghrelin, anti-IGF-1, and anti-GH). Standard, samples, and horseradish peroxidase (HRP) conjugated with anti-ghrelin, anti-IGF-1, and anti-GH were successively added, incubated, removed, and plates were washed thoroughly. Tetramethylbenzidine (TMB), the color development substrate, was turned into blue by HRP catalysis and then into yellow by acid. The hormone concentration in the samples and the color depth had a positive connection. The optical density values were read on a microplate spectrophotometer (Epoch 2, BioTek Instruments, Inc., Winooski, Vermont, USA) at a wavelength of 450 nm. The inter-and intra-assay coefficients of variation were 9% and 15%, respectively [26].

#### Total DNA extraction, library construction and sequencing

Metagenomic DNA from the cecal digesta was extracted using a QIAGEN DNeasy® PowerSoil® Pro Kit (Qiagen Ltd., Dusseldorf, North Rhine-Westphalia, Germany) according to the guidelines. A Rapid DNA Library Prep Kit (Beijing Huaruikang Technology Co., Ltd., China) was applied to perform metagenome library construction according to the manufacturer’s protocols. The library preparations were sequenced on a NovaSeq 6000 platform.

#### Metagenome assembly, binning, and taxonomic assignment

Metagenome assembly and binning were performed according to our previous study [28]. Briefly, assembly and binning were processed by MEGAHIT (v1.1.3), MaxBin2 (v2.2.6), Concoct (v1.0.0) and MetaBAT2 (v2.12.1), respectively. Only those metagenomes assembled genomes (MAGs) with a completeness ≥ 80% and contamination ≤ 10% were used for downstream analyses. GTDB-Tk was used for taxonomical annotation of MAGs. Functional profiling was performed using HUMAnN2 (v0.11.2).

#### Gene catalog construction and annotation

Gene prediction from the assembled contigs was performed by Prodigal (v2.6.3) [29]. MMseq2 was used to construct a nonredundant gene catalog, and gene clustering was carried out with 95% homology and 90% overlap [30]. High-quality reads of each sample were aligned against the gene catalog by BWA-MEM2 (V2.1) to calculate relative gene abundance [31]. We used KofamKOALA (v1.3.0) to annotate the non-redundant gene catalogs to assign KEGG Orthology (KOs) and KEGG module profiles [32]. The gene catalog was annotated by ABRicate software, which integrates the dbCAN2 database [33], the virulence factor database [34] and the Comprehensive Antibiotic Resistance Database [35] to profile virulence factors (VFs), antimicrobial resistance genes (ARGs), and CAZymes in the chicken gut microbiome.

### Microbial co-occurrence network construction

Co-occurrence networks were constructed using Spearman correlations and the associated *P*-values corrected for multiple comparisons with Benjamini-Hochburg adjustments. A valid co-occurrence event should be a robust correlation if the Spearman’s correlation coefficient was both > |0.7| and statistically significant at *P* < 0.05 [36] using the R package iGraph [37]. Network visualization was conducted using Gephi [38] and Cytoscape 3.5.1 [39]. We characterized network modularity (M) with threshold M > 0.4 to define modular structures [40].

### In vitro study

#### Anaerobic fermentation of AOS

Cecal digesta samples were collected from healthy broiler chickens. The collected fresh cecal digesta samples were immediately diluted (10% w/v) with 0.1 mol/L phosphate-buffered saline (pH = 7.0), centrifugated at 500 × *g* for 5 min in room temperature. Then the supernatant was used as inocula. The anaerobic fermentations of AOS were conducted according to the reported method with slight modification [41]. The culture medium ingredients are listed in Table S2. The 10 mL of the homogenized fecal slurry was inoculated in each vessel with a final inoculation of 1.0%. AOS solution (after passing-through a 0.22-μm sieve), which was mixed in an autoclaved medium to achieve final concentrations of 0, 0.01%, 0.02%, and 0.04% AOS. In parallel, medium without any carbon substances was used as control. After medium inoculation, the vessels were placed in an anaerobic cabinet at 37 °C for 48 h to maintain the anaerobic condition. The fermentation supernatant was used for SCFAs analysis as described above.

#### Pathogen inhibition assays

To evaluate the antibacterial ability of AOS, liquid cultures of *Escherichia coli* CAU0757, *Salmonella typhimurium* CMCC 50115, and *Clostridium perfringens* ATCC 13124 strains were harvested by centrifugation at 3,000 × *g* for 5 min, washed twice, resuspended, and diluted to an OD_600_ of 1.0 using PBS. A 100 μL aliquot of each bacterium was inoculated into 10 mL medium with AOS concentrations of 0, 0.02%, 0.2%, and 2% and incubated at 37 °C for 48 h. The samples were collected at 0, 6, 12, 24, and 48 h, and the OD_600_ of the aqueous phase was measured.

#### Effect of AOS on the growth of *Dorea* sp. CML553

To determine if *Dorea* sp. CML553 (a gift from Prof. Jie Feng at the Institute of Microbiology, Chinese Academy of Sciences) can utilize AOS to grow, a reduced-carbon-source medium based on GAM broth with the omission of glucose was used as a basal medium (BM, pH = 7.0 ± 0.2, Table S3). An aliquot of 100 μL of *Dorea* sp. CML553 culture from GAM was inoculated into 10 mL of BM + 0.3% glucose or AOS, BM + 1.5% glucose or AOS; the fermentation lasted for 24 h under anaerobic conditions. The growth of *Dorea* sp. CML553 was determined by measuring the OD_600_ values, and the supernatants were collected for SCFAs analysis.

### Statistical analysis

Statistical analysis was performed using SAS v. 9.4 (SAS Institute, Cary, NC, USA) with all data tested for normality. If the data were not normally distributed, non-parametric tests were used. Linear and quadratic polynomial contrasts were tested using the CONTRAST statement to analyze the BW, ADG, ADFI, and F/G. Data related to the growth of *Dorea* sp. CML553, SCFAs (in vitro), and pathogen inhibition assays were analyzed in a completely randomized design using the GLM procedures of SAS software. The data, including intestinal morphology, enzyme activities, serum parameters, SCFAs concentrations and the gene expression levels collected for quantitative parameters, were analyzed using Student’s *t* test. The cages served as the experimental unit. *P* < 0.05 represented statistically significant, and those with 0.05 ≤ *P* ≤ 0.10 tended toward significance. The R package vegan (v2.5–7) was used to calculate α-diversity, principal component analysis (PCA), non-metric multidimensional scaling (NMDS), and principal coordinate analysis (PCoA) based on the Bray–Curtis distance. Wilcoxon rank sum test and linear discriminant analysis effect size (LEfSe) were used for differential abundance analysis. The correlation between microbes and SCFAs was calculated by Spearman correlation with R package vegan (v2.5–7) under the correlation coefficient criterion of > |0.35|, and statistically significant at *P* < 0.05.

## Results

### Cloning, expression, and characterization of bacterial alginate lyases in *P. pastoris*

**Fig. 1:**
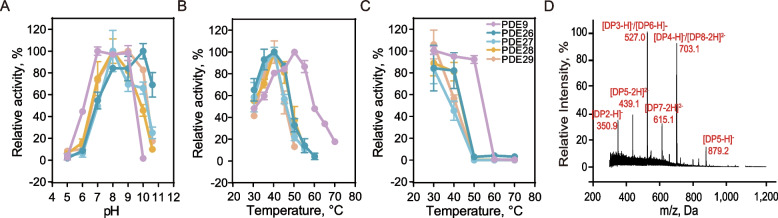
Biochemical characterization of the alginate lyases under standard assay conditions. (**A**) pH-optimum; (**B**) temperature-optimum; (**C**) effect of temperature on the thermostability; (**D**) mass spectrum of the reaction product from sodium alginate obtained by PDE9

To generate an enzymatic preparation of AOS, we first cloned and expressed five alginate lyases (PDE9, PDE26, PDE27, PDE28, and PDE29) from different bacteria in *P. pastoris* GS115. The molecular weights of the five proteins expressed in GS115 were estimated to be approximately 30–50 kDa by SDS‒PAGE analysis (Fig. 1A). The optimal pH values were determined to be pH 7 for PDE9, pH 10 for PDE26, pH 8 for PDE27 and PDE28, and pH 9 for PDE29 at 37 °C; the pH profiles for all enzymes displayed classical bell shapes (Fig. 1B). Under the optimal pH, PDE9 was more active at 50 °C, while PDE26, PDE27, PDE28, and PDE29 showed optimal enzymatic activity at 40 °C (Fig. 1C). PDE9 was stable below 50 °C and retained 92.4% of its activity after 1 h at 50 °C (Fig. 1C); however, PDE26 and PDE28 were more stable at 30 °C and 40 °C, and PDE27 and PDE29 were only stable at 30 °C. Under the optimal temperature and pH, the enzyme activities of PDE9, PDE26, PDE27, PDE28, and PDE29 were 220.9, 45.9, 34.1, 39.0 and 18.8 U/mL, respectively. These results indicated that alginate lyase PDE9 was expressed at relatively high yield and activity in *P. pastoris*, and the high thermostability and pH stability ensured its applications in preparing AOS in our study. HPLC‒MS analysis revealed that the degree of polymerization of the PDE9-generated AOS ranged from 2 to 8 (Fig. 1D).

### AOS enhances the growth performance and gut function of broiler chickens

To determine the effects of the prepared AOS on broiler chicken growth performance, 1-day-old birds were provided with 100, 200 and 400 mg/kg AOS in their diet for 42 d. We found that the final BW, ADG and ADFI of chickens were quadratically increased (*P* < 0.05) with increasing AOS levels (Table 2). The diet supplemented with 200 mg/kg AOS significantly increased the birds’ ADG and ADFI compared with the control and 400 mg/kg AOS groups during the whole production period (*P* < 0.05). However, no statistically significant differences were found for the F/G ratio among the groups (Table 2), indicating that dietary 200 mg/kg AOS increased the chicken feed intake and thus improved their BW and ADG. We then focused on revealing the effects of dietary 200 mg/kg AOS on chicken physiology and gut microbiota in the following analyses.

**Table 2:** Growth performance of broiler chickens fed graded concentrations of AOS

Items	Treatment				SEM	*P*-value		
	0	0.01%	0.02%	0.04%		Treatment	Linear	Quadratic
BW, g/bird								
d 21	665.94	643.43	642.93	606.59	20.626	0.261	0.056	0.930
d 42	2,502.98	2,619.07	2,746.66	2,533.98	67.986	0.075	0.841	0.013
ADG, g/d/bird								
d 1–21	31.14	30.02	29.99	28.17	1.031	0.261	0.056	0.930
d 22–42	88.73	94.25	100.33	90.20	3.237	0.075	0.841	0.013
d 1–42	59.52^b^	61.51^ab^	65.50^a^	58.13^b^	1.629	0.028	0.524	0.006
ADFI, g/d/bird								
d 1–21	47.56	46.65	46.84	44.77	1.122	0.357	0.093	0.747
d 22–42	151.98^b^	162.58^ab^	165.46^a^	151.44^b^	3.856	0.030	0.639	0.004
d 1–42	98.67^bc^	103.51^ab^	105.04^a^	96.99^c^	2.039	0.028	0.343	0.004
F/G								
d 1–21	1.53	1.56	1.56	1.60	0.025	0.335	0.077	0.856
d 22–42	1.72	1.73	1.65	1.69	0.036	0.439	0.471	0.431
d 1–42	1.66	1.69	1.63	1.68	0.026	0.443	0.889	0.446

^a–c^Means with different superscripts in the same row differ (*P* < 0.05, *n* = 8)

**Fig. 2:**
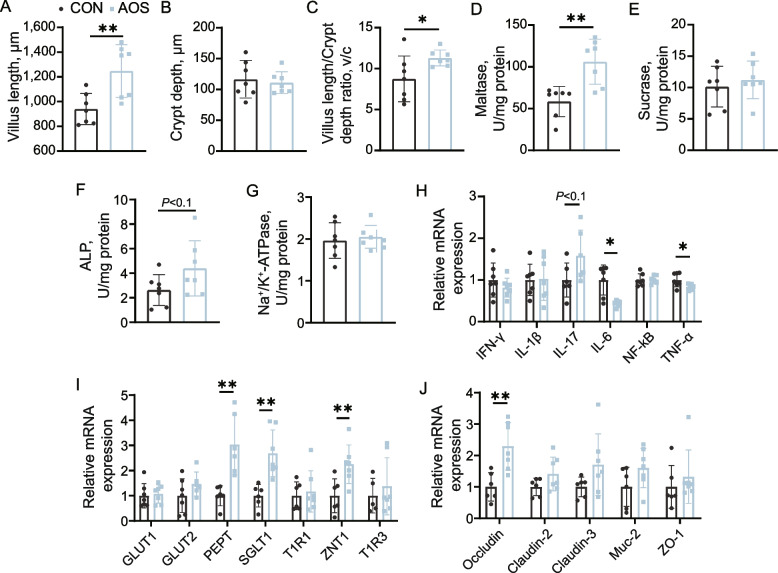
AOS increases the gut function of broiler chickens. Jejunum gut morphometrics: (**A**) villus length, (**B**) crypt depth, and (**C**) villus length/crypt depth ratio; The (**D**) maltase, (**E**) sucrase, (**F**) ALP, and (**G**) Na^+^/K^+^-ATPase activities in the small intestine of broiler chickens supplemented with or without AOS; Relative gene expression of (**H**) cytokines, (**I**) nutrient transport-related genes, and (**N**) tight junction proteins of broiler chickens fed diets containing AOS. Data are expressed as the mean ± SD (*n* = 8). ^*^P < 0.05, ^**^P < 0.01

Birds treated with 200 mg/kg AOS showed 32.69% and 29.03% increases in intestinal villus length and villus/crypt ratio, respectively, in the jejunum compared to the control birds (*P* < 0.05), while the crypt depth was not affected (Fig. 2A–C, Fig. S2). Of note, the maltase activities of AOS-fed birds in the jejunum mucosa were significantly greater than those of the control birds (*P* < 0.05, Fig. 2D), and the jejunum mucosa ALP activities tended to be increased at d 42 (*P* < 0.1, Fig. 2F). The relative expression levels of the nutrient transport-related genes *SGLT1*, *PEPT* and *ZNT1* were profoundly higher (*P* < 0.05) in the AOS-fed birds than those on the control birds (Fig. 2I), and the expression of occludin, which is involved in maintaining intestinal integrity, was also higher at d 42 (*P* < 0.05, Fig. 2J). Additionally, in cecal tonsils from AOS-fed birds, cytokine expression was marked by the upregulation of the cytokine interleukin-17 (IL-17, *P* < 0.1) and the downregulation of tumor necrosis factor-α (TNF-α) and IL-6 (*P* < 0.05) at d 42 (Fig. 2H), suggesting that in-feed AOS modulated chicken immunity.

### AOS increases growth-related hormones and SCFAs

**Fig. 3:**
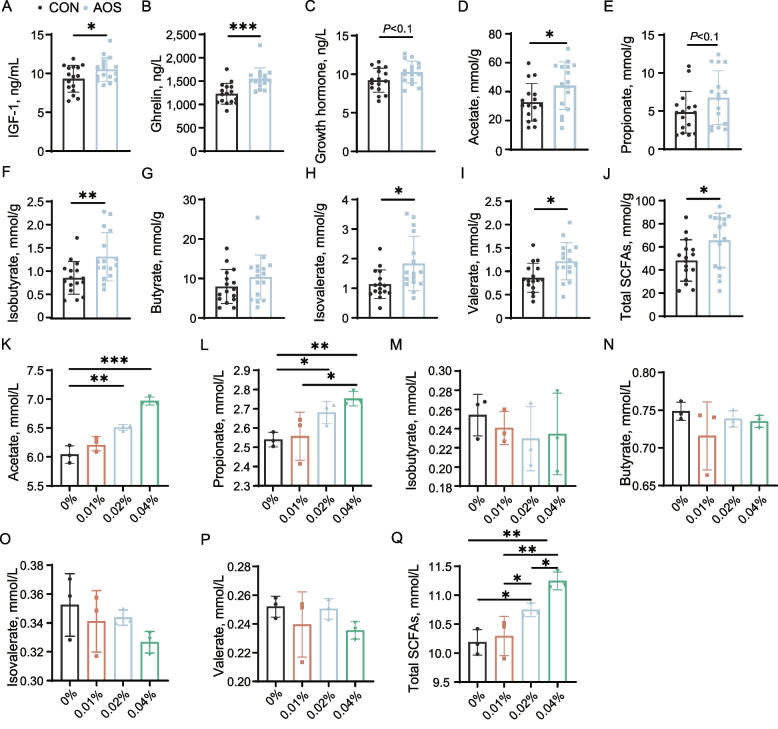
AOS increases growth-related hormones and SCFAs. Serum (**A**) IGF-1, (**B**) ghrelin and (**C**) GH activity (*n* = 16); Cecal concentrations of (**D–J**) SCFAs of broiler chickens (*n* = 16). The (**K–Q**) SCFAs production in batch cultures of AOS using the chicken cecal contents (*n* = 3). Data are expressed as the mean ± SD. ^*^P < 0.05, ^**^P < 0.01

Since AOS improved chicken growth performance, we wondered if there were changes in growth-related hormones in chicken serum. Interestingly, we found that the amounts of ghrelin and IGF-1 were higher for birds fed diets with AOS compared with the control (*P* < 0.05), and GH levels also displayed an increasing trend (*P* < 0.1, Fig. 3A–C). As SCFAs are known to influence host hormones [42], we examined SCFAs levels in the chicken gut and found that the amounts of acetate, valerate, isobutyrate, isovalerate and total SCFAs were all markedly increased in the cecal digesta (*P* < 0.05, Fig. 3D–J), which may hint that AOS stimulated the growth of SCFA-producing bacteria in the chicken gut. To confirm the ability of AOS to promote the gut microbiota to produce SCFAs, in vitro batch fermentation of AOS using chicken cecal contents was performed. We found that the production of both acetate, propionate and the total SCFA increased (*P* < 0.05) in an AOS-dose-dependent manner (Fig. 3K–Q).

### AOS changes the chicken cecal microbial community structure

**Fig. 4:**
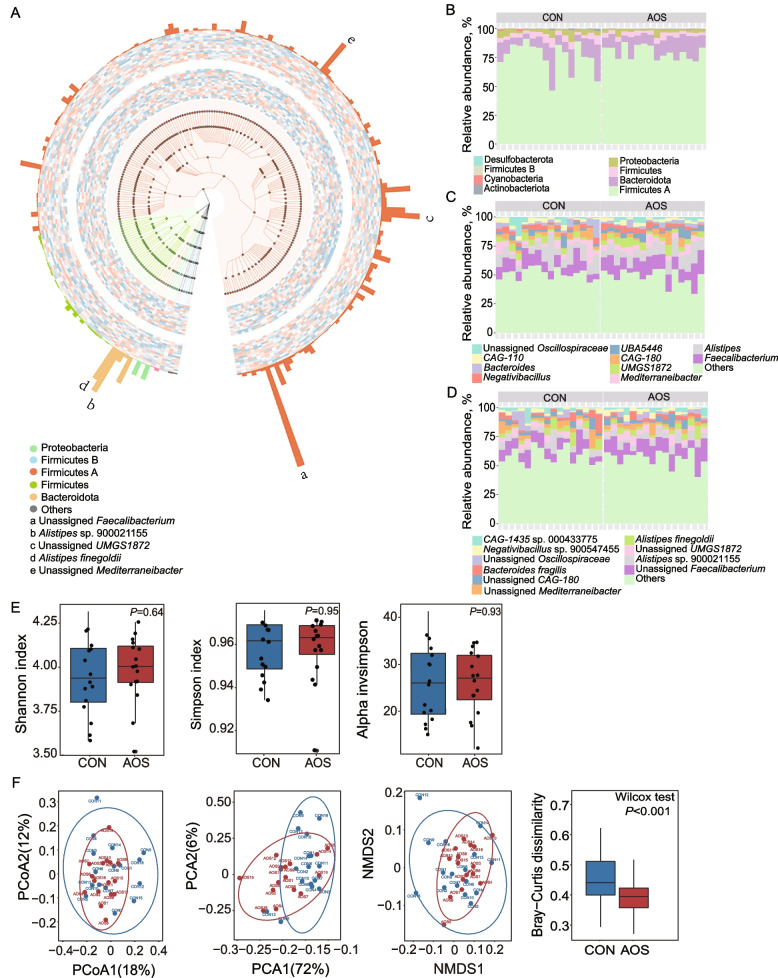
AOS changes cecal microbial community structure. (**A**) Taxonomic classification tree of 220 MAGs at the species level using GraPhlAn. From the inner to outer rings, the first and second ring represents the CON and AOS group, respectively. The height of each bar in the third ring represents the relative abundance of each specie. Relative abundance of major bacteria (**B**) phyla, (**C**) genus, and (**D**) species for all individuals (*n* = 16). (**E**) Microbiome α-diversity in AOS and control groups at specie-level (*n* = 16). (**F**) PCoA, PCA, NMDS, and Bray–Curtis dissimilarity of intestinal microbial community structure (*n* = 16)

To investigate the impacts of AOS on the chicken gut microbiota, we sequenced the metagenomes of 32 chicken cecal samples (16 control vs. 16 AOS samples) by shotgun sequencing. The metagenomes were assembled, binned and taxonomically classified. Metagenome binning yielded 986 metagenome-assembled genomes (MAGs) of > 80% completeness and < 10% contamination (Table S4). In total, we found 8 bacterial phyla having relative abundance > 0.1% in more than 50% of samples, with Firmicutes A, Bacteroidota, Firmicutes, Proteobacteria and Actinobacteriota being the top 5 most abundant phyla in the chicken gut microbiota (Fig. 4A–B). At the species level, Unassigned *Faecalibacterium* was the most abundant species in the cecal microbiota, followed by *Alistipes* sp. 900021155, Unassigned *UMGS1872*, *Alistipes finegoldii*, and Unassigned *Mediterraneibacter* (Fig. 4A and D, Table S5). There were no differences in the Shannon, Simpson or InvSimpson diversity indices between the AOS and control samples (Fig. 4E, S3B). However, the within-group Bray‒Curtis distance-based PCA, PCoA and NMDS plots showed that the two groups were separated from each other (Fig. 4F), indicating that the beta-diversity of the two groups was significantly different. We also used the MetaPhlAn2 pipeline for taxonomic profiling (Fig. S3A), the results of which were consistent with those obtained with the binning method (Fig. S3C), and confirmed that AOS altered the overall cecal microbiome community structure of chickens.

### AOS increases the modularity of gut microbial interactions

**Fig. 5:**
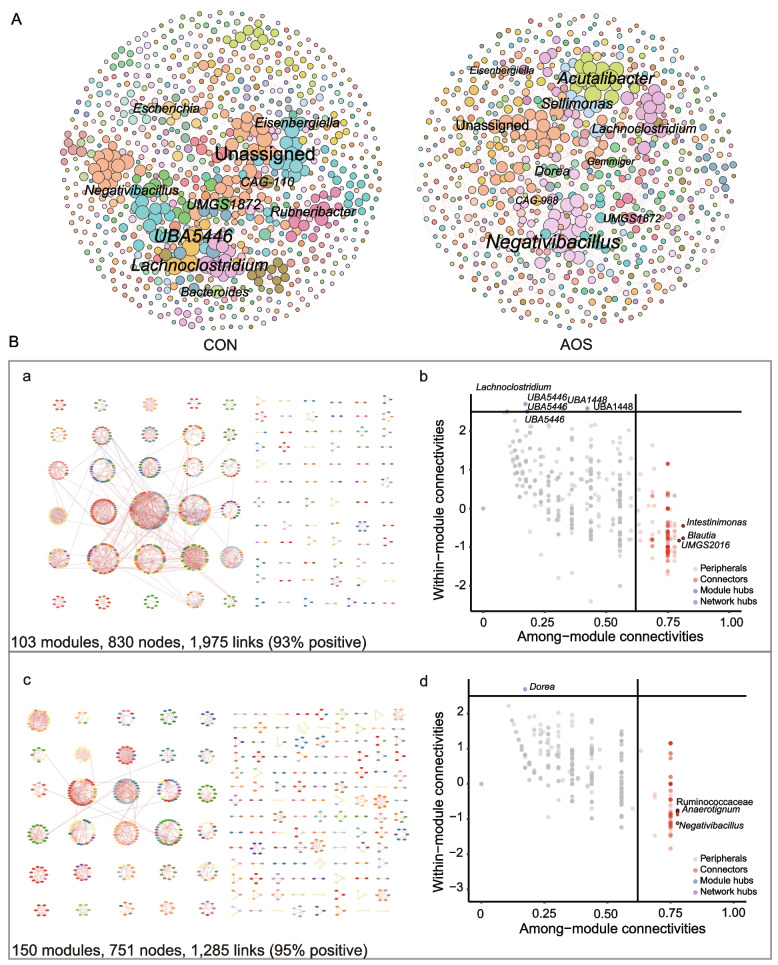
Microbial co-occurrence networks, modularity, and keystone taxa. **A** Co-occurrence networks of 986 MAGs in the CON and AOS groups (*n* = 16). Different colors of nodes indicate different genera. The node size is weighted based on node degree. The red and blue links represent positive and negative correlations, respectively. Highly connected modules within (**B**a) CON and (**B**c) AOS networks (*n* = 16). Classification of nodes to identify putative keystone taxa within (**B**b) CON and (**B**d) AOS networks (*n* = 16)

We then explored the effects of AOS on the microbial interactions in the chicken cecal microbiota through MAG-based co-occurrence network analysis (Fig. 5A). The networks in the control and AOS groups displayed different topological features (Fig. S3D). In total, the empirical networks consisted of 830 nodes with 1,975 edges (a mean of 4.03 edges per node) for the CON network and 751 nodes with 1,285 edges (a mean of 2.64 edges per node) for the AOS network (Fig. 5Ba, c). The average clustering coefficient (ACC), average path distance (APL), and modularity in the two networks were higher than those in the corresponding randomized networks (Fig. S3D), suggesting that the constructed networks had typical hierarchical, small-world, and modular characteristics. We next compared the topological structures of the two networks. The APLs between all paired nodes were 9.45 edges with a diameter of 23 edges in the AOS network and 8.94 edges with a diameter of 32 edges in the CON network, and the ACCs were 0.59 and 0.71 in the CON network and the AOS networks, respectively (Fig. S3D). Both networks contained modules with modularity values ≥ 0.87 (Fig. S3D), and of note, 150 modules with high modularity were observed in the AOS network, while only 103 were found in the CON network (Fig. 5Ba, c). The taxa tended to co-occur (positive correlations) rather than co-exclude (negative correlations), and positive correlations accounted for 93% and 95% of the potential interactions observed in the CON and AOS networks, respectively (Fig. 5Ba, c).

To identify the possible topological roles of taxa in the co-occurrence networks, we classified the nodes into four categories (module hubs, network hubs, connectors, and peripherals), based on their within-module connectivity (Zi) and among-module connectivity (Pi) values [40]. No network hubs were identified in the two networks, but connectors and module hubs, regarded as key taxa in network topology, were found in both networks (Fig. 5Bb, d). *Lachnoclostridium*, two taxa from the genus *UBA1448*, and three taxa from the genus *UBA5446* were classified as module hubs (Fig. 5Bb), and *Intestinimonas*, *Blautia* and *UMGS2016* were identified as connectors in the CON network. The majority of the nodes in the AOS network were peripherals, with most of their links inside their modules. The genus *Dorea* was identified as the only module hub, and *Negativibacillus*, *Anaerotignum*, and Ruminococcaceae were connectors in the AOS network (Fig. 5Bd). Overall, the microbial interactions in the AOS network seemed to be simplified but were highly modularized.

### AOS alters chicken cecal microbial community function

**Fig. 6:**
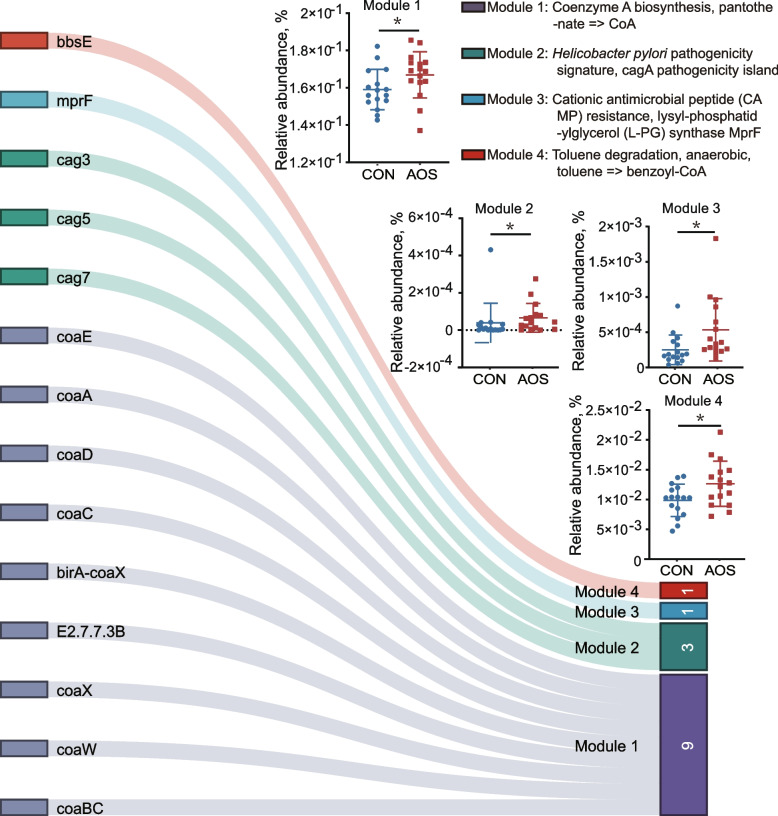
The KEGG pathway modules enriched in the AOS group. The upper right part showed the four KEGG pathway modules with significant differences between the control group and the AOS group (*n* = 16), and the lower left part showed the KO genes enriched in the module pathway

We next constructed a 1.6 million nonredundant gene catalog using metagenomic sequencing data and compared the abundances of metabolic pathways and carbohydrate-active enzymes between the CON and AOS groups. KEGG annotation analysis showed that four KEGG pathway modules, "Coenzyme A biosynthesis, pantothenate", "*Helicobacter pylori* pathogenicity signature, cagA pathogenicity island", "Cationic antimicrobial peptide (CAMP) resistance, lysyl-phosphatidylglycerol (L-PG) synthase MprF", and "Toluene degradation, anaerobic, toluene", were significantly overrepresented in the AOS-fed birds (*P* < 0.05, Fig. 6). A total of nine genes belonging to the coenzyme A biosynthesis (CoA) module were enriched in the AOS group, and the abundances of four genes encoding the key enzymes (EC2.7.1.33, EC4.1.1.36, EC2.7.7.3, and EC6.3.2.5) of the pantothenate and CoA biosynthesis pathways were significantly higher in this group (Fig. S4A). Furthermore, a total of 14 differentially abundant pathways were found between the two groups (*P* < 0.05) by using the HUMAnN2 analysis pipeline, among which six pathways, including THISYN PWY (superpathway of thiamin diphosphate biosynthesis I), PWY 6834 (spermidine biosynthesis III), and SER GLYSYN PWY (superpathway of *L*-serine and glycine biosynthesis I), were enriched in the AOS group, and eight pathways, such as PWY0 1338 (polymyxin resistance) and PWY0 1533 (methylphosphonate degradation I), were enriched in the CON group (Fig. S4B).

For the CAZyme profile, a total of 352 genes encoding family-level CAZymes were identified in the two groups. The top 10 most abundant CAZyme-encoded genes are shown in Fig. S6A. CAZymes from the glycoside hydrolase (GH) (CON vs. AOS, 56.85% vs. 56.50%) and glycosyltransferase (GT) (CON vs. AOS, 14.98% vs. 15.15%) classes were predominant in the chicken cecal microbiota; the families GH13 and GT2 in the GH and GT classes, respectively, displayed the highest abundance (Fig. S5A, Table S6). Dietary AOS did not increase the overall abundance of CAZymes but enriched PL7, PL9_1, PL9_2, PL15_1, and PL17 family enzymes compared with the CON group (*P* < 0.05, Fig. S5B). In the CAZyme database, alginate lyases mainly belong to seven PL families: PL5, 6, 7, 14, 15, 17, and 18; the enrichment of PL7, PL15, and PL17 family enzymes in the AOS group may imply that AOS promoted the growth of gut microbes that can utilize AOS or alginate.

### Differentially abundant gut microbes driven by AOS

**Fig. 7:**
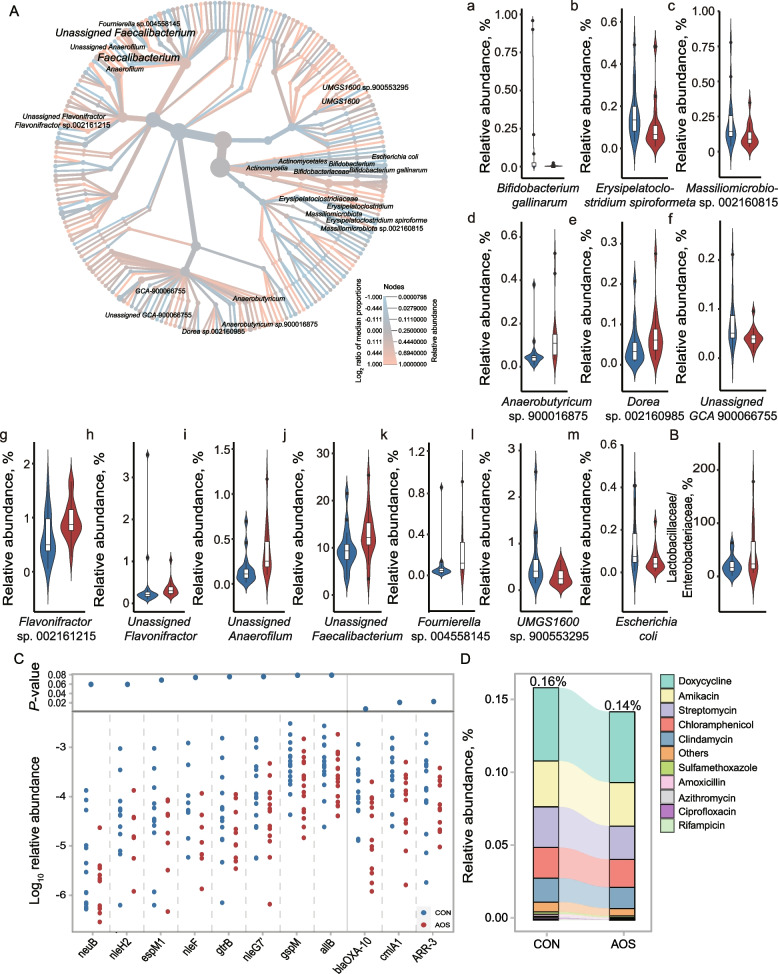
Differential enrichment of microbes in the CON and AOS groups. **A** Cladogram of significantly different taxa identified in the chicken cecal microbial community of CON (blue) and AOS (red) based on the cut-off of LDA > 2.0 and *P* < 0.05 (*n* = 16). Dots represent taxonomic hierarchies. **B** Comparison of the Lactobacillaceae/Enterobacteriaceae ratio (*n* = 16). **C** Differentially abundant VFs genes and ARGs in the two groups (*n* = 16). **D** Comparison of the ARG category abundance (*n* = 16)

We next explored which microbes in the chicken cecal microbiota were affected by dietary AOS. The LEfSe results showed that 13 species were differentially abundant between the two groups (LDA > 2.0 and* P* < 0.05). AOS significantly increased the abundance of *Anaerobutyricum* sp. 900016875, *Dorea* sp. 002160985, *Flavonifractor* sp. 002161215, Unassigned *Anaerofilum*, Unassigned *Faecalibacterium*, and *Fournierella* sp. 004558145; bacteria from the genera *Anaerobutyricum* [43], *Dorea* [4] and *Faecalibacterium* [44] are all known to be SCFA producers. Meanwhile, AOS decreased the abundances of *Bifidobacterium gallinarum*, *Erysipelatoclostridium spiroforme*, *Massiliomicrobiota* sp. 002160815, Unassigned *GCA* 900066755, Unassigned *Flavonifractor*, *UMGS1600* sp. 900553295, and *Escherichia coli* (Fig. 7A). As *Lactobacillus* bacteria are representatives of beneficial microbes, while Enterobacteriaceae is usually regarded as harmful, a ratio of Lactobacillaceae to Enterobacteriaceae has been used as a determinant of intestinal health [45]. We found that AOS significantly increased this ratio in the cecal microbiota of chickens, suggesting an improvement of the gut environment after AOS intervention (Fig. 7B).

Pathogenic bacteria such as those in the Enterobacteriaceae are known to be sources of virulence factors and ARGs [28]. We found that the relative abundances of eight virulence genes, *neuB*, *nleH2*, *espM1*, *nleF*, *gtrB*, *nleG7’*, *gspM*, and *allB*, were significantly lower in the AOS-fed birds than in the control birds (*P* < 0.05, Fig. 7C, Table S7). All these genes were previously found to be carried by bacteria of the Enterobacteriaceae family. Additionally, a total of 91 ARGs were detected in the chicken cecal microbiota. The AOS-fed birds had lower relative abundance of ARGs than the CON group (CON vs. AOS, 0.16% vs. 0.14%; *P* = 0.08) (Fig. 7D, Table S8). The abundances of *blaOXA-10*, *cmlA1*, and *ARR-3*, conferring resistance to rifampicin, amoxicillin, and chloramphenicol, respectively, were significantly decreased in the AOS group (Fig. 7C). Taken together, these results suggested that dietary AOS promoted the growth of SCFA-producing bacteria and decreased the abundance of pathogens such as those in Enterobacteriaceae as well as the virulence genes and ARGs they carried.

To determine if AOS can inhibit pathogenic bacteria, an in vitro inhibitory test was performed using *S. typhimurium*, *E. coli*, and *C. perfringen*s as targets, all of which are important pathogens that cause chicken gut infections. The results showed that all three AOS concentrations (0.02%, 0.2% and 2%) inhibited the growth of the three pathogens in a dose-dependent manner, and the inhibitory effect of AOS on *S. typhimurium* and *C. perfringen*s was stronger than that on *E. coli* (Fig. S5C), confirming the role of AOS in inhibiting chicken gut pathogens.

### The abundance of *Dorea* sp. positively correlates with the concentrations of SCFAs and growth-related hormones

**Fig. 8:**
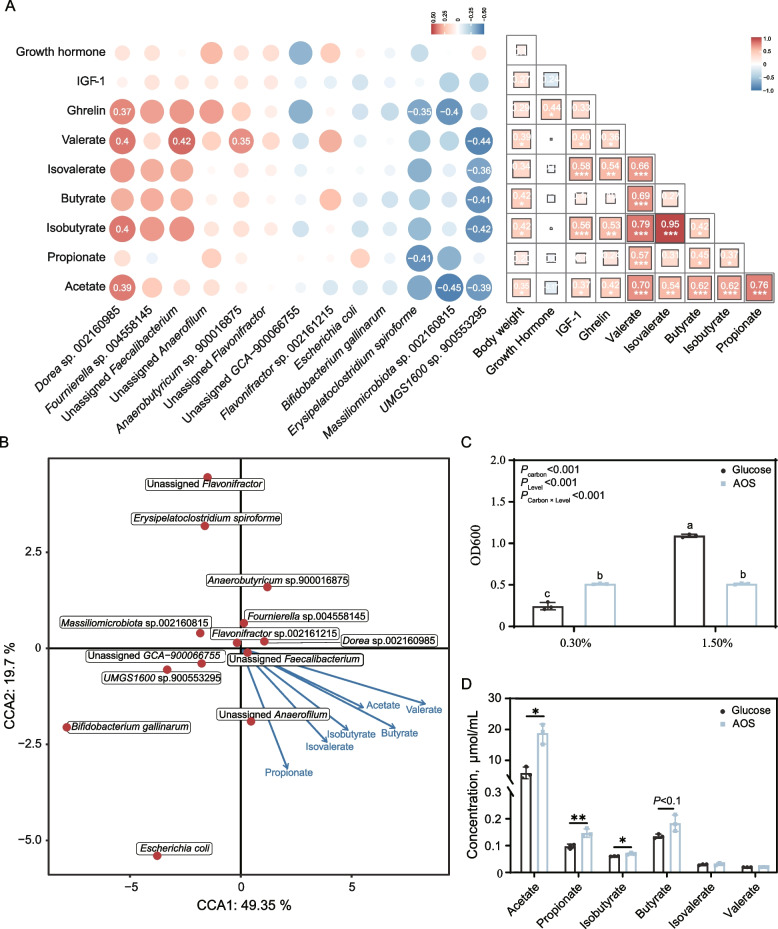
The abundance of *Dorea* sp. correlates with the concentrations of SCFAs and growth-related hormones. **A** The sizes of the squares or circles are scaled according to the correlation degree. The intensities of blue and red colors indicate values of positive and negative correlation coefficients, respectively (*n* = 16). **B** CCA between species and SCFAs (*n* = 16). **C** In vitro growth of *Dorea* sp. CML553 on graded concentrations of AOS and glucose (*n* = 3). **D** SCFAs production in batch cultures containing 0.3% AOS or glucose (*n* = 3). Data are expressed as the mean ± SD. ^*^P < 0.05, ^**^P < 0.01

We then performed correlation analysis to identify the relationships between the differentially abundant cecal microbes and the SCFA levels, chicken growth performance and growth-related hormones. Interestingly, we found that AOS-enriched *Dorea* sp. 002160985 was positively correlated with the concentrations of ghrelin, valerate, isobutyrate and acetate (*P* < 0.05), while *UMGS1600* sp. 900553295 showed negative correlations with most SCFAs, and both *E. spiroforme* and *Massiliomicrobiota* sp. 002160815 were negatively correlated with ghrelin (Fig. 8A). We further analyzed the relationships among body weight, GH, IGF-1, ghrelin and SCFAs. Specifically, acetate, isobutyrate, butyrate and valerate were all positively correlated with body weight, and acetate displayed significantly positive associations with propionate, isobutyrate, butyrate, isovalerate, valerate, ghrelin and IGF-1 (Fig. 8A). These results indicated that SCFAs, especially acetate, may exert strong positive effects on chicken hormones and growth performance.

We next performed a Canonical correlation analysis to reveal the associations between the differentially abundant cecal microbial species and the concentrations of the SCFAs. The results also showed that *Dorea* sp. 002160985 was positively associated with SCFAs such as acetate, propionate, and butyrate (Fig. 8B). Considering that *Dorea* is the only module hub in the microbial interaction network of the AOS group, as mentioned above, we speculated that this bacterium played a critical role in generating SCFAs and in modulating growth-related hormones in AOS-fed birds.

### AOS can be utilized by *Dorea* sp. to grow and to produce SCFAs

We then performed an in vitro fermentation experiment to investigate whether species from the *Dorea* genus could utilize AOS to grow and to produce SCFAs. A basic nitrogen medium was prepared with glucose (0.30% and 1.50%) or AOS (0.30% and 1.50%) added as the sole carbon source for the cultivation of *Dorea* sp. CML553*.* Compared with glucose, a relatively low concentration of AOS (0.3%) significantly promoted the growth of *Dorea* sp. CML553, while a high concentration (1.5%) displayed an inhibitory effect (Fig. 8C, Table S9). The SCFAs produced by *Dorea* sp. CML553 mainly consisted of acetate but also included propionate, butyrate, and isobutyrate, and 0.3% AOS markedly increased the production of the first three SCFAs compared with 0.3% glucose. Of note, the production of acetate in AOS medium was nearly three times higher than that in the glucose medium (18.78 μmol/mL vs. 5.93 μmol/mL, Fig. 8D). A further analysis of the genomes of seven representative species in the *Dorea* genus confirmed the presence of four key genes involved in the acetate synthesis pathway in this genus, i.e., *ackA*, *pta*, *grdB*, and *pflB* genes encoding acetate kinase, phosphate acetyltransferase, glycine reductase complex selenoprotein B, and formate C-acetyltransferase, respectively (Table S10).

## Discussion

A large number of alginate lyases have been characterized, mainly applying *E. coli* as an expression host [46]. Herein, we expressed alginate lyases from different bacterial sources and compared their biochemical characteristics by using the *P. pastoris* expression system, which is regarded to have significant advantages in the production of recombinant proteins [47]. Importantly, compared with *E. coli*, *P. pastoris* secretes recombinant soluble proteins into the medium and can be used to produce giant quantities of enzymes by high-density fermentation without complicated purification steps [48]. Among the five alginate lyases we characterized in this study, PDE9 showed the highest enzyme activity and better characteristics when expressed in *P. pastoris*. Compared with its counterpart expressed in *E. coli* (Alg7D) [14], the optimal pH of the enzyme increased from pH 7 to 9, and the thermal stability increased from 42% enzyme activity after 30 min to 92.4% after 1 h of incubation at 50 °C. The improvement of these performances is probably due to the posttranslational modifications of the enzyme protein in *P. pastoris*, including disulfide bond formation and glycosylation, which are critical for the structural stability and enzyme activity of recombinant proteins [49, 50].

We showed that the inclusion of PDE9-enzymatically produced AOS in broiler feed significantly improved chicken growth performance. Similar growth-promoting effects of AOS have also been found in other animals, such as weaned pigs [8] and grass carp [11], suggesting that AOS is a beneficial additive in animal production. However, in contrast to our results, Yan et al. [13] reported that enzymatically prepared AOS had no obvious impact on the growth performance of broiler chickens. One of the major reasons may be the relatively high dose of AOS (0.04% and 0.2%) used in their study. We showed that the performance of the birds displayed a quadratic response to the concentrations of AOS, with the optimal dose of 0.02%. A high level of AOS may induce a strong mucosal immune response at the cost of decreasing chicken growth performance [13].

The improvement of chicken growth performance after AOS supplementation was found to be accompanied by or due to the promotion of chicken gut functions reflected by the intestinal morphology and associated enzyme activities, barrier function, and immunity. AOS significantly increased the villus/crypt ratio and villus length in the chicken jejunum, which may help increase the absorption surface of the intestine and provide a favorable environment for nutrient uptake. Accordingly, the increased activity of intestinal mucosal maltase, an enzyme in the small intestinal brush border, is essential for the degradation of disaccharides into monosaccharides and facilitates absorption [51]. Additionally, AOS tended to increase the activity of ALP, which was enterocyte differentiation-dependent and was considered to be an important marker enzyme for major digestive and absorption functions in the small intestine [52]. Moreover, AOS increased the expression of occludin, which is related to intestinal barrier integrity, and decreased the mRNA expressions of the pro-inflammatory cytokines TNF-α and IL-6, which can directly or indirectly damage intestinal epithelial cells [53].

Interestingly, we found that AOS significantly increased the concentrations of the growth-related hormones ghrelin, GH, and IGF-1 in chicken serum. The GH/IGF-1 signaling pathway is known to play a key role in regulating chicken growth and BW [54]; the GH part of the somatotropic axis is the main regulator of growth rate, while IGF-1 may regulate both growth rate and BW. Moreover, GH and IGF-1 can stimulate tissue growth by regulating the metabolisms of protein, carbohydrate, and lipid [54], and the growth-promoting effect of GH is partly mediated by circulating or locally produced IGF-1 [55]. In chickens, it has been reported that plasma GH concentrations increase in response to elevated ghrelin levels [56, 57], and the increased level of endogenous ghrelin also acts as a hunger signal to induce appetite [58, 59]. We thus propose that, in addition to improving gut function, the growth-promoting effect of AOS in broiler chickens may also be mediated by the increase in endogenous ghrelin and the resulting regulation of GH/IGF-1 signals and/or promotion of chicken appetite.

Dietary fibers cannot be digested by host enzymes but are metabolized by microbiota in the cecum and colon and thus change the gut microbial community structure and function [60]. The gut microbiota and its metabolic products are known to take part in promoting chicken growth and gut health [61]. We showed that dietary AOS altered the chicken cecal microbiome community structure and increased the modularity of the microbial interaction network (Fig. 5B). Modularity is an evolved property that could enhance the flexibility of the generation of various phenotypes during development in cellular networks [62], resulting in the production of more functional units. The high modularity could provide an advantage for improving the stability of bacterial networks and helping bacterial communities survive when exposed to rapid environmental changes [63]. Moreover, modularity allows different taxa to function independently with less overlap and connectivity among bacterial taxa [64]. We therefore proposed that AOS may enhance the stability of the microbial interaction and benefit the gut microbiota in coping with microbial factors destroying the community structure.

Additionally, AOS changed the topological roles of taxa in the networks; species from SCFA producers such as *Negativibacillus*, *Anaerotignum*, and Ruminococcaceae were identified as network connectors (i.e., nodes linking different modules together), and a species from the genus *Dorea* was a module hub in the AOS network. Connectors and module hubs in the microbial interaction network may act as keystone taxa in maintaining network structure stability [64]. Furthermore, consistent with previous findings in weaned pigs [65] and broilers [13], we demonstrated that AOS inhibited the growth of pathogens such as those from Enterobacteriaceae both in vivo and in vitro and decreased the abundance of microbial VF genes and ARGs in the chicken gut. Bacteria from the family Enterobacteriaceae are among the most common pathogens in gut infection [66]. A decrease in Enterobacteriaceae by dietary AOS may contribute to the reduced inflammatory responses and the improved intestinal mucosal barrier function we observed.

Due to the changes in the chicken gut microbiota structure, the metabolic potentials of the gut microbial community were accordingly altered by dietary AOS. Metagenome-based functional analysis revealed that AOS increased gut microbial biosynthesis, including the synthesis of vitamins, amino acids, spermidine, and especially CoA. As an ubiquitous and essential cofactor in all living cells, CoA acts as a carrier for activated acyl groups and carbonyl-activating group in different metabolic and energy-producing reactions [67], resulting in a diverse range of metabolically active thioester derivatives, including acetyl-CoA, malonyl-CoA and HMG-CoA (3-hydroxy-3-methylglutaryl-CoA) [68]. Therefore, the increase in the CoA biosynthesis pathway in AOS-fed birds may indicate an elevation of gut microbial metabolic activity, such as processing dietary fiber, which may help the bird better extract dietary energy and nutrition. Moreover, CoA is the main intracellular carrier of organic acids, including SCFAs, branched chain acids and long-chain fatty acids [69]. Consistent with this, our in vivo and in vitro targeted metabolomic analyses of SCFAs indicated that both the levels of acetate and total SCFAs significantly increased after AOS supplementation.

SCFAs are important fuels for intestinal epithelial cells (IECs) and regulate the function of IECs through different mechanisms including modulating cell proliferation and differentiation [70], impacting absorption and transport of nutrients [71], strengthening gut barrier functions [70], and regulating immune response [72]. For example, propionate and butyrate increased intestinal glucose production and transport [73, 74], and butyrate inhibited the expression of TNF-α, IL-1 and IL-6 to exert anti-inflammatory effects [75]. Therefore, the increased level of gut SCFAs that improved the gut function may be a critical reason for the better performance of chickens fed AOS in this study. Additionally, SCFAs are known to act as signaling molecules to regulate host physiological metabolism, such as regulating intestinal gluconeogenesis and fat deposition [76]. Recently, Perry et al. [77] found that gut microbe-derived acetate can stimulate ghrelin secretion and lead to obesity; other studies have shown that circulating ghrelin levels changed when the microbiome was altered [78]. Therefore, the increased acetate production in the chicken gut microbiota by dietary AOS prompted us to suggest its role in promoting ghrelin secretion and thus contributing to chicken growth. Consistent with this hypothesis, significantly positive associations between acetate and ghrelin and IGF-1 and BW were observed. Importantly, we demonstrated that AOS could effectively promote the growth of acetate-producing *Dorea* species and that species from *Dorea* were the sole module hub taxon in the AOS network. Together, these results established the connections among AOS, chicken gut microbiota/SCFAs, growth hormone signals and chicken growth performance and highlighted the importance of the genus *Dorea* in regulating chicken growth.

## Conclusion

In conclusion, we demonstrated that the enzymatically produced AOS effectively promoted broiler chickens’ daily gain and feed intake. The mechanisms involved in the growth-promoting effects of AOS are concluded as follows. On the one hand, AOS modulates the chicken gut microbiota, promotes the growth of SCFA producers and increases the level of SCFAs in the chicken gut. The increase in SCFAs lowers gut pH, restricts the growth of pathogenic bacteria (which may also be attributed to a direct action of the AOS itself), regulates intestinal immunity, promotes gut barrier function, and improves gut digestion and absorption function. On the other hand, AOS increases the growth of acetate producers, especially *Dorea* species, and the increase in acetate activates the parasympathetic nervous system, which in turn promotes ghrelin secretion. Ghrelin promotes the secretion of GH through the GH secretagogue receptor, which in turn increases the synthesis and secretion of IGF-1; GH and/or IGF-1 promotes the growth of chicken muscle. The increase in endogenous ghrelin also stimulates chicken appetite and thereby increases chicken feed intake. These factors may contribute collectively to the improved chicken growth performance.

## Supplementary Information

**Additional file 1: Fig. S1.** SDS-PAGE of recombinant PDE9, PDE26, PDE27, PDE28, and PDE29. **Fig. S2.** Histological evaluation of intestinal tissues (× 40) after exposure to AOS. **Fig. S3.** AOS changes the chicken cecal microbial community structure revealed by MetaPhlAn2. **Fig. S4.** AOS alters microbiota function. **Fig. S5.** AOS alters chicken cecal microbial community function and inhibits pathogenic bacteria. **Table S1.** Forward and reverse primers for quantitative PCR. **Table S2.** The fermentation medium contained the following constituents (per liter). **Table S3.** The GAM broth contained the following constituents (per liter).

## Data Availability

The datasets (Table S4–S10) supporting the conclusions of this article are available in the figshare (https://doi.org/10.6084/m9.figshare.21174037). All metagenomes have been deposited and are available at the NCBI Sequence Read Archive under accession BioProject PRJNA873123.

## Abbreviations

AOS: Alginate oligosaccharide
ADG: Average daily gain
ADFI: Average daily feed intake
ALP: Alkaline phosphatase
ACC: Average clustering coefficient
APL: Average path distance
ARGs: Antimicrobial resistance genes
F/G: Feed/gain ratio
GH: Growth hormone
GH: Glycoside hydrolase
GT: Glycosyltransferase
IGF-1: Insulin-like growth factor-1
IL-17: Interleukin-17
IL-6: Interleukin-6
MAGs: Metagenome-assembled genomes
PL: Polysaccharide lyase
SCFA: Short-chain fatty acid
TNF-α: Tumor necrosis factor-α
VFs: Virulence factors
