# Supplementary material for: Enzymatically prepared alginate oligosaccharides improve broiler chicken growth performance by modulating the gut microbiota and growth hormone signals
Source: J Anim Sci Biotechnol. 2023 Jul 3;14:96. doi: 10.1186/s40104-023-00887-4 (PMC10316573; doi:10.1186/s40104-023-00887-4)
Supplement: Supplementary file 1 — Additional file 1: Fig. S1. SDS-PAGE of recombinant PDE9, PDE26, PDE27, PDE28, and PDE29. Fig. S2. Histological evaluation of intestinal tissues (× 40) after exposure to AOS. Fig. S3. AOS changes the chicken cecal microbial community structure revealed by MetaPhlAn2. Fig. S4. AOS alters microbiota function. Fig. S5. AOS alters chicken cecal microbial community function and inhibits pathogenic bacteria. Table S1. Forward and reverse primers for quantitative PCR. Table S2. The fermentation medium contained the following constituents (per liter). Table S3. The GAM broth contained the following constituents (per liter). [file 40104_2023_887_MOESM1_ESM.docx]

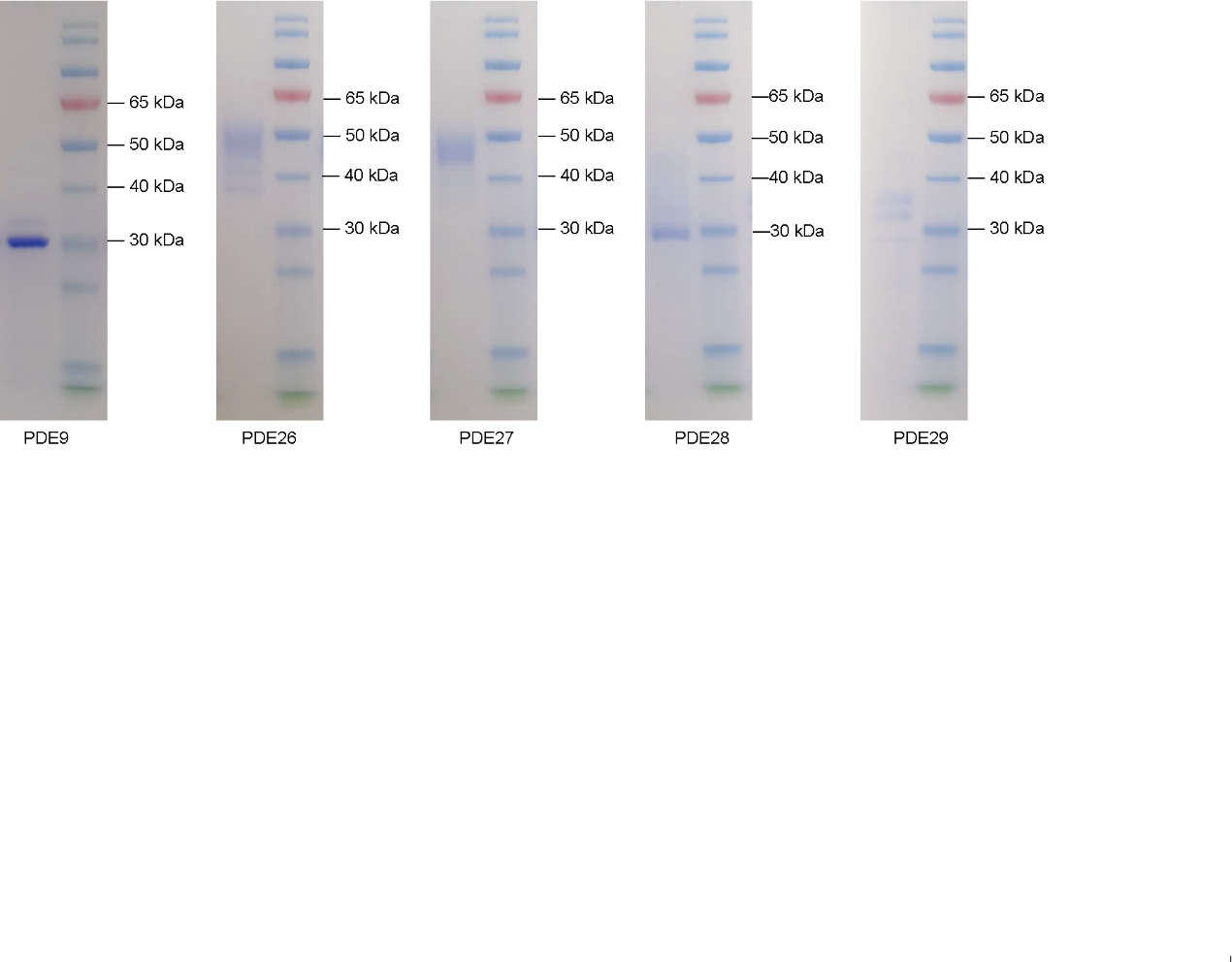


**Fig. S1** SDS-PAGE of recombinant PDE9, PDE26, PDE27, PDE28, and PDE29


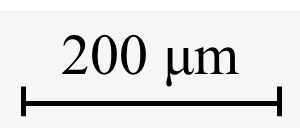

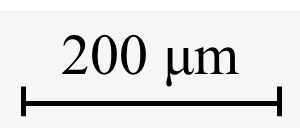

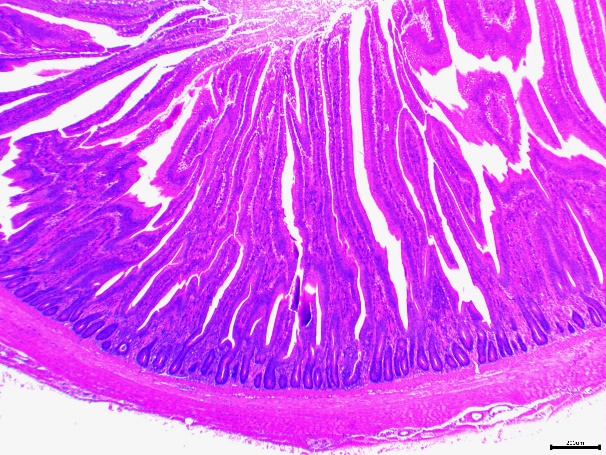

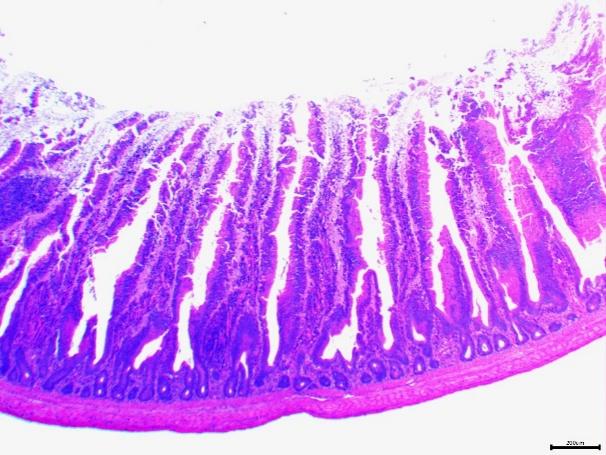


CON

AOS

**Fig. S2** Histological evaluation of intestinal tissues (×40) after exposure to AOS. Scale bar is 200 μm


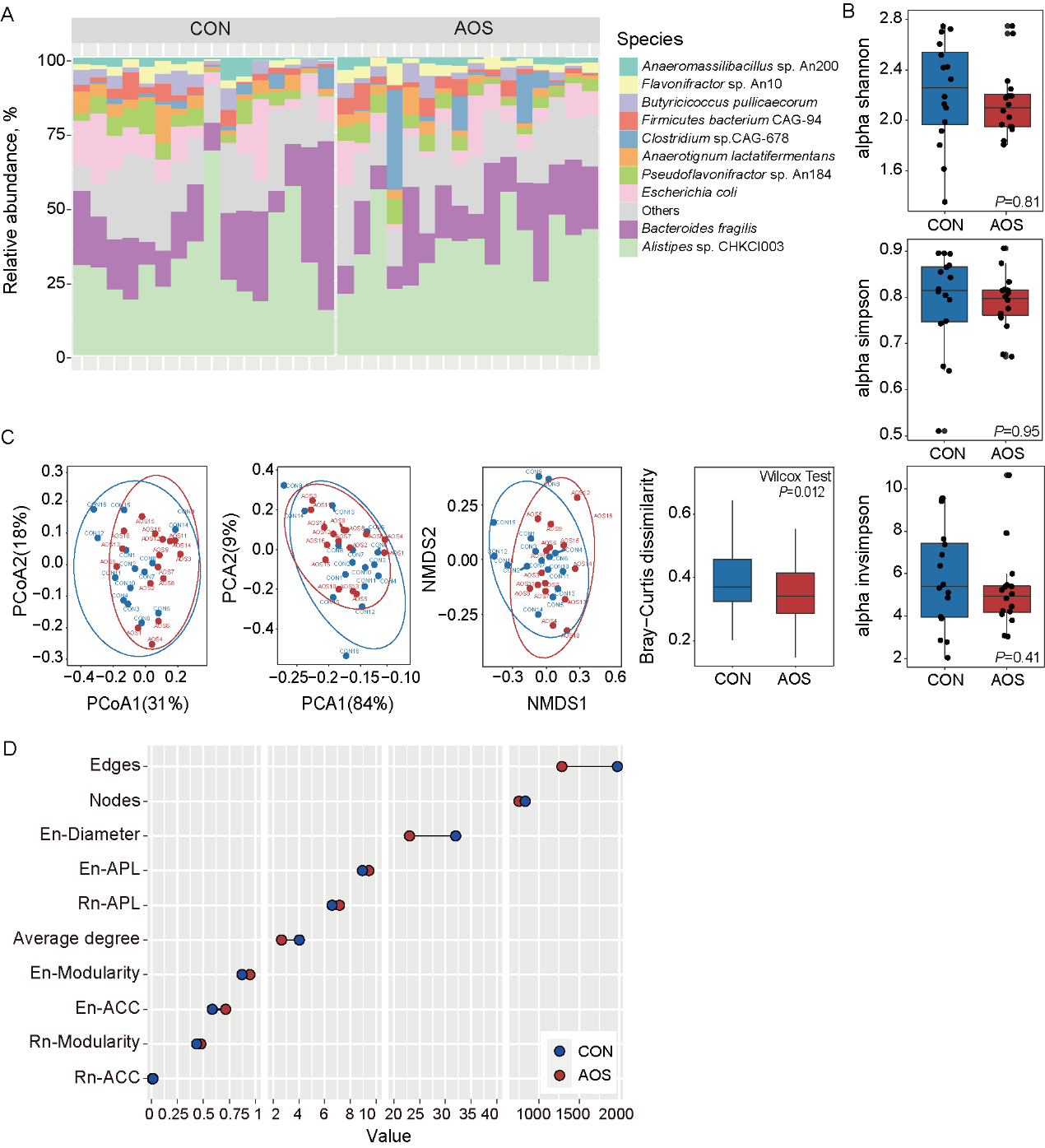


**Fig. S3** AOS changes the chicken cecal microbial community structure revealed by MetaPhlAn2. **A** Relative abundance of major bacterial species (relative abundance > 0 1% in more than 90% animals). **B** Microbiome α-diversity in AOS and control groups at specie-level (*n* = 16). **C** PCoA, PCA, NMDS, and Bray-Curtis dissimilarity calculated from the species abundance (*n* = 16). **D** Empirical and randomized molecular ecology network (MENs) properties of microbial communities under different treatment (*n* = 16). Randomized networks were performed by rewiring all the nodes and links corresponding to empirical networks 1000 times. En: Empirical network. Rn: Randomized networks. ACC: Average clustering coefficient. APL: Average path distance

***
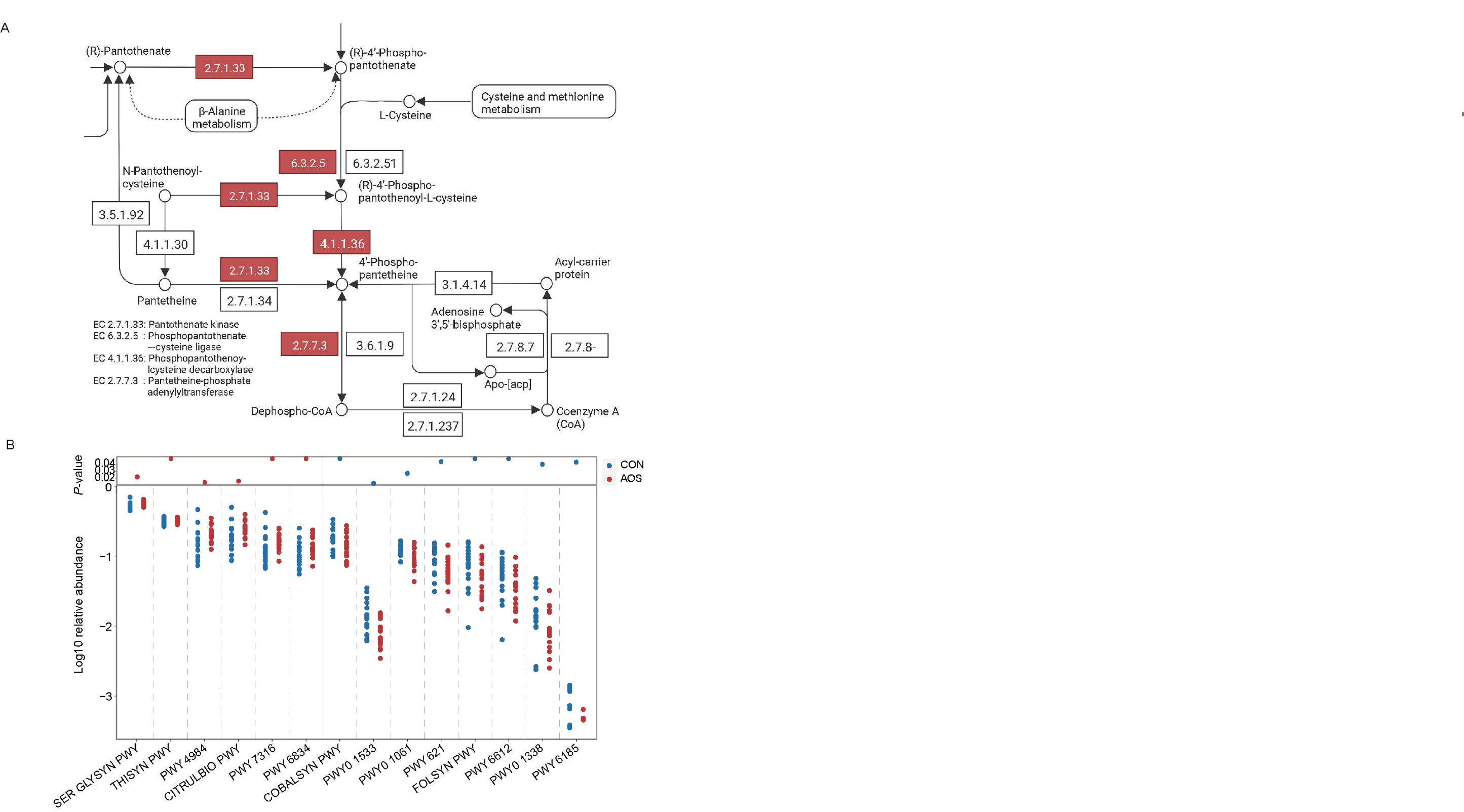
*****Fig. S4** AOS alters microbiota function. **A** Metabolic pathway to produce CoA and enzymes enriched by AOS. **B** MetaCyc hierarchy of pathway classifications showed different metabolic pathways enriched in the CON and AOS groups (*n* = 16). SER GLYSYN PWY: superpathway of L-serine and glycine biosynthesis I; THISYN PWY; superpathway of thiamin diphosphate biosynthesis I; PWY 4984: urea cycle; CITRULBIO PWY: L-citrulline biosynthesis; PWY 7316: dTDP-N-acetylviosamine biosynthesis; PWY 6834: spermidine biosynthesis III; COBALSYN PWY: adenosylcobalamin salvage from cobinamide I; PWY0 1533: methylphosphonate degradation I; PWY0 1061: superpathway of L-alanine biosynthesis; PWY 621: sucrose degradation III (sucrose invertase); FOLSYN PWY: superpathway of tetrahydrofolate biosynthesis and salvage; PWY 6612: superpathway of tetrahydrofolate biosynthesis; PWY0 1338: polymyxin resistance; PWY 6185: 4-methylcatechol degradation (ortho cleavage)

*
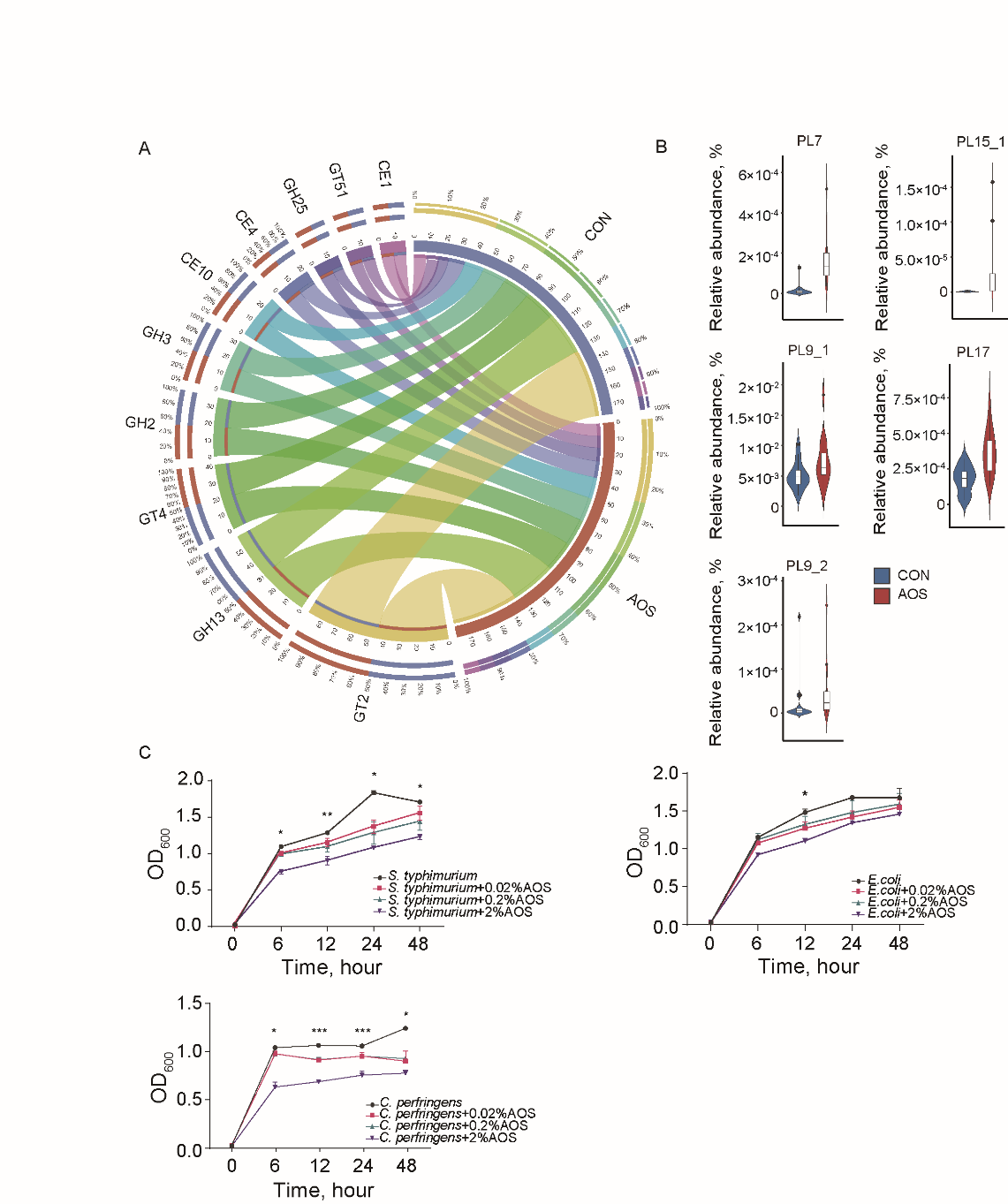
*

**Fig. S5** AOS alters chicken cecal microbial community function and inhibits pathogenic bacteria. **A** The gut microbial CAZyme gene profile (*n* = 16). **B** Differentially abundant CAZyme genes between the two groups (n = 16; *P* < 0.05). GHs: glycoside hydrolases; PLs: polysaccharide lyases; GTs: glycosyltransferase; CEs: carbohydrate esterases. **C** In vitro growth of *Salmonella typhimurium, Escherichia coli, and Clostridium perfringens* on graded concentrations of AOS (*n* = 3)

**Table S1** Forward and reverse primers for quantitative PCR

| **Gene** | **Accession no.** | **Primer sequence (5′→3′) Accession no.** |
| --- | --- | --- |
| *β-actin* | L08165 | F：GAGAAATTGTGCGTGACATCA |
|  |  | R：CCTGAACCTCTCATTGCCA |
| *Occludin* | GI:464148 | F：ACGGCAGCACCTACCTCAA |
|  |  | R：GGGCGAAGAAGCAGATGAG |
| *ZO-1* | XM_413773 | F：CTTCAGGTGTTTCTCTTCCTCCTC |
|  |  | R：CTGTGGTTTCATGGCTGGATC |
| *Claudin-2* | NM_001277622.1 | F：CTGCTCACCCTCATTGGA |
|  |  | R：AACTCACTCTTGGGCTTCTG |
| *Claudin-3* | NM_204202.2 | F：CCAAGATCACCATCGTCTCC |
|  |  | R：CACCAGCGGGTTGTAGAAAT |
| *Mucin-2* | XM_421035 | F：TTCATGATGCCTGCTCTTGTG |
|  |  | R：CCTGAGCCTTGGTACATTCTTGT |
| *IL-1β* | NM_204524 | F：ACTGGGCATCAAGGGCTA |
|  |  | R：GGTAGAAGATGAAGCGGGTC |
| *IL-6* | AJ250838 | F：GCTCGCCGGCTTCGA |
|  |  | R：GGTAGGTCTGAAAGGCGAACAG |
| *IL-17* | NM_204460.1 | F：TATCAGCAAACGCTCACTGG |
|  |  | R：AGTTCACGCACCTGGAATG |
| *NF-kB* | NM_205134.1 | F：TGGAGAAGGCTATGCAGCTT |
|  |  | R：CATCCTGGACAGCAGTGAGA |
| *TNF-α* | NM_204267 | F：GAGCGTTGACTTGGCTGTC |
|  |  | R：AAGCAACAACCAGCTATGCAC |
| *IFN-γ* | NM_205149.1 | F：AGCTGACGGTGGACCTATTATT |
|  |  | R：GGCTTTGCGCTGGATTC |
| *GLUT1* | NM_205209.1 | F：TCCTCCTGATCAACCGCAAT |
|  |  | R：TGTGCCCCGGAGCTTCT |
| *GLUT2* | Z22932 | F：CACACTATGGGCGCATGCT |
|  |  | R：ATTGTGCCTGGAGGTGTTGGT |
| *PepT1* | NM_204365.1 | F：CCCCTGAGGAGGATCACTGTT |
|  |  | R：CAAAAGAGCAGCAGCAACGA |
| *SGLT1* | NM_001293240 | F：GCCATGGCCAGGGCTTA |
|  |  | R：CAATAACCTGATCTGTGCACCAGTA |
| *ZnT1* | XM_421021.5 | F：TCCGGGAGTAATGGAAATCTTC |
|  |  | R：AATCAGGAACAAACCTATGGGAAA |
| *T1R1* | XM_425734.4 | F：GTGTCATCCCCACAACCAA |
|  |  | R：CACCACTGCCTCAAAGAAGG |
| *T1R3* | XM_425740.3 | F：CATTACCGTCTTCGCCACTC |
|  |  | R：CTCTGTTCAAATCGGGCTTC |

*β -Actin* = Beta-actin; *IL-1β =* interleukin 1 beta; *IL-6 =* interleukin 6; *IL-17 =* interleukin 17; *NF-kB* = nuclear factor kappa B; *TNF-α* = tumor necrosis factor alpha; IFN-γ = interferon gamma; *GLUT1* = glucose transporter 1; *GLUT2* = glucose transporter 2; *PepT1* = Peptide transporter-1; *SGLT1* = sodium-glucose transporter 1; *ZnT1 =* Zinc transporter; *T1R1* = taste receptor type 1 member 1; *T1R3* = taste receptor type 1 member 3

**Table S2** The fermentation medium contained the following constituents (per liter)

| **Ingredient, /L** | **In vitro fermentation** |
| --- | --- |
| Tryptone, g | 2.00 |
| Micromineral solution^1^, μL | 125.00 |
| Buffer solution^2^, mL | 250.00 |
| Micromineral solution^3^, mL | 250.00 |
| *L*-cysteine HCl, g | 0.40 |
| Resazurin solution, mL | 1.00 |

^1^Micromineral solution (per liter): 132 g CaCl_2_·2H_2_O; 100 g MnClּ_2_·4H_2_O; 10 g CoCl_2_·6H_2_O; FeCl_3_·6H_2_O

^2^Buffer solution (per liter): 4 g NH_4_CHO_3_; 35 g NaHCO_3_

^3^Micromineral solution (per liter): 5.7 g Na_2_HPO4; 6.2 g KH_2_PO_4_; 0.6 g MgSO_4_·7H_2_O

**Table S3** The GAM broth contained the following constituents (per liter)

| **Ingredient, /L** | **GAM** |
| --- | --- |
| Proteose peptone, g | 15.00 |
| Casein peptone, g | 10.00 |
| Soy protein, g | 3.00 |
| Yeast extract, g | 5.00 |
| Beef extract, g | 2.00 |
| Digested serum, g | 13.50 |
| Beef liver extract, g | 1.20 |
| Potassium dihydrogen phosphate, g | 2.50 |
| Sodium chloride, g | 3.00 |
| Soluble starch, g | 0.30 |
| Cysteine-HCl, g | 0.30 |
| Sodium thioglycolate, g | 0.15 |
